# The Glucan-Remodeling Enzyme Phr1p and the Chitin Synthase Chs1p Cooperate to Maintain Proper Nuclear Segregation and Cell Integrity in *Candida albicans*

**DOI:** 10.3389/fcimb.2019.00400

**Published:** 2019-11-22

**Authors:** Genny Degani, Laura Popolo

**Affiliations:** Department of Biosciences, University of Milan, Milan, Italy

**Keywords:** cell wall assembly, β-(1,3)-glucanosyltransferases, septum, nuclear segregation, cell integrity, morphogenesis

## Abstract

GH72 family of β-(1,3)-glucanosyltransferases is unique to fungi and is required for cell wall biogenesis, morphogenesis, virulence, and in some species is essential for life. *Candida albicans PHR1* and *PHR2* are pH-regulated genes that encode GH72 enzymes highly similar to Gas1p of *Saccharomyces cerevisiae. PHR1* is expressed at pH ≥ 5.5 while *PHR2* is transcribed at pH ≤ 5.5. Both are essential for *C. albicans* morphogenesis and virulence. During growth at neutral-alkaline pH, Phr1p-GFP preferentially localizes to sites of active cell wall formation as the incipient bud, the mother-daughter neck, the bud periphery, and concentrates in the septum at cytokinesis. We further investigated this latter localization. In *chs3*Δ cells, lacking the chitin of the chitin ring and lateral cell wall, Phr1p-GFP still concentrated along the thin line of the primary septum formed by chitin deposited by chitin synthase I (whose catalytic subunit is Chs1p) suggesting that it plays a role during formation of the secondary septa. RO-09-3143, a highly specific inhibitor of Chs1p activity, inhibits septum formation and blocks cell division. However, alternative septa are produced and are crucial for cell survival. Phr1p-GFP is excluded from such aberrant septa. Finally, we determined the effects of RO-09-3143 in cells lacking Phr1p. *PHR1* null mutant was more susceptible to the drug than the wild type. The *phr1*Δ cells were larger, devoid of septa, and underwent endomitosis and cell death. Phr1p and Chs1p cooperate in maintaining cell integrity and in coupling morphogenesis with nuclear division in *C. albicans*.

## Introduction

*Candida albicans*, a major human fungal pathogen, is a polymorphic fungus endowed of extraordinary morphological plasticity and adaptive capacity. The fight against fungal invasive infections still relies on a small repertoire of drugs, among which the inhibitors of the synthesis of β-(1,3)-glucan (echinocandins) target the fungal cell wall. It is urgent to develop new drugs, or combination of drugs, to counteract the rise of drug resistance and the limited efficacy echinocandins have toward some fungal species such as *Aspergillus fumigatus*.

The fungal cell wall is formed by β-(1,3) and β-(1,6)-glucans, mannoproteins and a tiny amount of chitin, and has the mechanical strength necessary to withstand the high turgor pressure of yeast cells. At the neck most of the bound chitin is cross-linked to β-(1,3)-glucan, the most abundant polysaccharide, whereas in lateral cell walls the attachment of chitin to β-(1,6)-glucan predominates and chitin of the primary septum is free (Cabib and Arroyo, 2013).

The enzymes of the GH72 family are responsible for β-(1,3)-glucan elongation and branching, crucial for the formation of the core of the cell wall and of the high molecular weight glucan-chitin polymer present at the bud neck region in yeast cells (Cabib et al., 2012; Aimanianda et al., 2017). Among the five GH72 encoding genes present in *C. albicans (PHR1, PHR2, PHR3, PGA4*, and *PGA5)* only two, *PHR1* and *PHR2*, appear to encode active enzymes that are anchored to the plasma membrane through a glycosyl-phosphatidylinositol and, in minor amount, are also cross-linked to the cell wall. *PGA4* encodes an inactive enzyme, *PHR3* and *PGA5* transcript level is very low or undetectable and Pga5p has anomalous sequence features (reviewed in Popolo et al., 2017). *PHR1* expression is triggered at external pH values ≥5.5 whereas *PHR2* is expressed at pH ≤5.5. Phr1p and Phr2p act on cell wall remodeling in the growing areas and in the septum both in yeast and hyphal form and, as expected, these enzymes have different pH optimum that mirrors the pH-dependent transcription pattern. Remarkably, β-(1,3)-glucan is shielded by an outer layer of mannoproteins that facilitate the escape of the pathogen from the immune cells (Hopke et al., 2016).

In unicellular yeasts, cell wall biogenesis requires a unique set of enzymes that are strictly regulated to maintain a tight coordination between growth and the discontinuous events of the cell cycle: bud emergence, DNA synthesis, mitosis and cell division. The end of the cell cycle is marked by cytokinesis and division of the septum wall, an essential process. Septation has been extensively studied in budding yeast (Cabib, 2004; Roncero and Sanchez, 2010) and the key enzyme in this process is the plasma membrane chitin synthase II (the catalytic subunit of which is *Sc*Chs2p). This enzyme is synthesized at early mitosis and reaches a maximum at the end of the M phase. It is targeted to the neck during mitosis exit. After execution of its function, *Sc*Chs2p is removed by endocytosis and degraded (Oh et al., 2012; Chin et al., 2016). The chitin disk synthesized by *Sc*Chs2p, named primary septum (PS), defines the plane along which septum abscission will occur and is covered on both sides by the secondary septa, presumably composed of the same material of lateral cell walls. Cell separation requires the help of the chitinases and endo-glucanases *Sc*Cts1p and *Sc*Eng1p, both localized to the daughter side of the septum (Baladron et al., 2002; Cabib, 2004). At cell separation, the secondary septa, the chitin ring and the PS remains on the mother cell forming the bud scar.

In *C. albicans*, septum formation is similar to *S. cerevisiae* and initiates with the synthesis of the chitin ring by recruitment of Chs3p at the site of bud emergence and is completed in G2 by Chs1p, the catalytic subunit of chitin synthase I and the equivalent of *Sc*Chs2p. Chs1p deposits chitin in the invagination that is created between the cell wall and the plasma membrane in concomitance with the closure of the plasma membrane by the cortical actomyosin contractile ring (Bulawa et al., 1995; Mio et al., 1996). As chitin is deposited centripetally, the PS is covered on both sides by cell wall material forming the secondary septa (Roncero and Sanchez, 2010). Cell separation is promoted by Cht3p and Eng1p, the equivalent of *Sc*Cts1p and *Sc*Eng1p, respectively (Dunkler et al., 2005; Esteban et al., 2005). Interestingly in *C. albicans*, the bud scar has a portion of exposed β-(1,3)-glucan (not covered by mannoproteins) that is recognized by Dectin-1 (Gantner et al., 2005). Since cleavage of septa is inhibited in hyphae, β-(1,3)-glucan remains hidden and this promotes the escape from the immune system.

*C. albicans* Chs1p is an essential enzyme required for PS formation but also for cell integrity (Munro et al., 2001). Other non-essential chitin synthases are Chs3p, Chs2p and Chs8p (Lenardon et al., 2010). Chs3p contributes to the majority of cell wall chitin which is deposited at the chitin ring and lateral walls, in response to a weakening of the cell wall and in the remedial septum. Chs2p and Chs8p are responsible for chitin in the septum and in the remedial septum (Walker et al., 2013; Preechasuth et al., 2015). In response to a pre-treatment with Calcofluor White/calcium chloride that stimulates chitin synthesis, the arrest of PS formation by use of a potent and highly specific inhibitor of Chs1p activity (RO-09-3143), activates the synthesis of remedial septa that are produced by the other active chitin synthases, i.e., Chs3p, Chs2p, and Chs8p, or in *chs3*Δ cells by Chs2p and Chs8p (Walker et al., 2013) and in some conditions restore cell division. This indicates that *C. albicans* possesses redundant salvage pathways to overcome the effects of the inhibition of primary septum formation.

Little is known about the role of β-(1,3)-glucan remodeling enzymes of GH72 family at the septum region. In this work, we deepened the study on the localization of Phr1p in the septum and investigated the impact of glucan remodeling on septum formation. By a chemo-synthetic approach we prove that Phr1p and Chs1p cooperate to maintain cell integrity and proper nuclear segregation.

## Methods

### Strains and Growth Conditions

The *Candida albicans* strains used in this work were CAF3-1 (*ura*3Δ::*imm*434/*ura3*Δ::*imm434*) and CAS8 (*ura*3Δ::*imm*434/*ura3*Δ::*imm434 phr1*Δ::*his*G/*phr1*Δ), kindly provided by Prof. W.A. Fonzi (Saporito-Irwin et al., 1995). The two strains expressing Phr1p-GFP, JC94-2 (Hopke et al., 2016) and *chs3*Δ-Phr1p-GFP (this work) harbor one allele of *PHR1* and two copies of *PHR1-GFP* the second of which is on the CIp20 plasmid (*PHR1/PHR1-GFP* CIp20-*PHR1-GFP*). An homozygous *chs3*Δ/*chs3*Δ strain derived from the Ura^−^ CAI4 strain was kindly provided by Dr. Mio (Mio et al., 1996) (*chs3*Δ::*hisG*/*chs3*Δ::*hisG*). The first copy of *PHR1-GFP* was obtained by a C-terminal internal tagging of GFP in the *PHR1* cds. The nucleotide sequence encoding GFP was inserted between the amino acids G489 and G490 of Phr1p by using a PCR-based strategy (Ragni et al., 2011). The second copy of *PHR1-GFP* was obtained by integration of the *StuI*-linearized CIp20-*PHR1-GFP* at the *RP10* locus (*PHR1/PHR1-GFP* CIp*20-PHR1-GFP*; Hopke et al., 2016). *C. albicans* cells were routinely grown at 25 or 30°C in YPD (10 g of yeast extract, 20 g of Bacto-peptone, 20 g of glucose, 25 mg of uridine per liter). The experiments were carried out in YPD-150 mM HEPES [4-(2-Hydroxyethyl) piperazine-1-ethanesulfonic acid sodium salt] buffered at the desired pH before sterilization. Growth was monitored as the increase in optical density at 600 nm (OD_600_). To induce hyphal development we used a protocol previously described (Degani et al., 2016). Briefly, blastospores were obtained by prolonged growth in YPD buffered at pH 6 and then transferred to M199-150 mM HEPES buffered at pH 7.5 at 37°C.

### Broth Microdilution and XTT Assays

Susceptibility of *C. albicans cells* to RO-09-3143 was tested by microdilution assay according to the CLS1 guidelines. Inoculum size was 10^5^ cells/ml. Cells exponentially growing at 30°C in YPD-150 mM HEPES, pH 8 were harvested by centrifugation and suspended in fresh medium at 2 × 10^5^ cells/ml. In a 96-well microplate, 100 μl of the cell suspension were added to an equal volume of medium containing different concentrations of RO-09-3143 (kindly donated by Roche) dissolved in DMSO (from 0.012 to 25 μM). All determinations were made in quadruplicate. Control wells contained only DMSO. The plates were incubated at 30°C and inspected at 24 h and 48 h. The effect of the treatment was evaluated by reading the turbidity with a Tecan Infinite F200 PRO microplate reader. For the XTT assay, the plate was centrifuged and wells were washed twice with 200 μl PBS. Then, 100 μl of 1 mg/ml XTT solution in PBS containing 1 μM menadione, dissolved at 1 M in acetone, were added to each well. Cells were suspended and the plate was incubated in the dark at 37°C for 1–2 h before reading the absorbance at 490 nm.

### Growth and Viability in Yeast and Hyphal Forms in the Presence of the Chs1p Inhibitor

To test the effect on growth in yeast form, exponentially growing cells in YPD-150 mM HEPES buffered at pH 8 at 30°C with shaking at 200 rpm, were collected by centrifugation and inoculated in pre-warmed fresh medium at a cell density of ~10^6^ cells/ml (~0.1 A_600_). After 30 min of equilibration, RO-09-3143 [10 μM, a concentration reported as non-lethal for the wild type (Sudoh et al., 2000)] or an equal volume of DMSO were added and cells were monitored at 2.5, 5, and 24 h.

To test the effect of the drug during induction of hyphal growth, blastospores of CAF3-1 and CAS8 were obtained as described at section Strains and Growth Conditions. Blastospores were suspended in pre-warmed M199-pH 7.5 at 2 × 10^6^ cells/ml and the culture was split in two: one received only DMSO and the other RO-09-3143 at 10 μM. At 0, 1, 3, 5, and 24 h after the shift, the formation of hyphae was monitored.

The effect on viability was assessed by methylene blue staining (Degani et al., 2016).

### Microscopy

Cells were routinely observed by phase-contrast microscopy after mild sonication consisting in two cycles of 6 s. To maximize Phr1p-GFP expression, cells were grown in YPD-150 mM HEPES buffered at pH 8. For GFP and Calcofluor white (CF), a specific dye for chitin, conventional and confocal microscopy was performed without fixation (Ragni et al., 2011). For the staining of nuclear DNA, cells were processed as previously described (Ragni et al., 2011). Cells with 1, 2, or more nuclei were counted using double beam (bright field and UV filter) to visualize the hyphal compartments and nuclei at the same time.

## Results

### Altered Localization of Phr1p-GFP in the Presence of Inhibition of Primary Septum Formation

**Figure 1:**
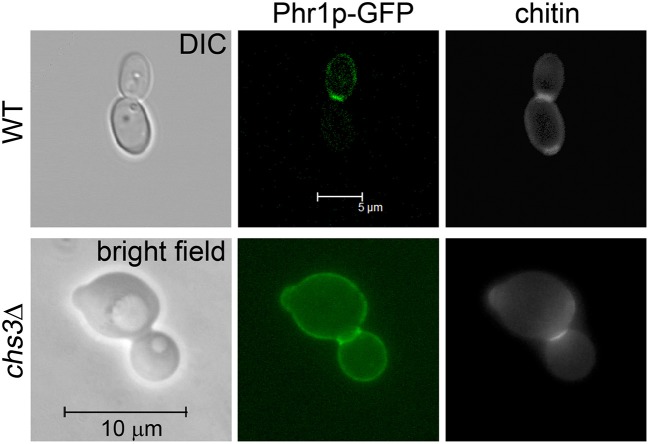
Phr1p-GFP localization at the septum in cells at cytokinesis. Wild type cells (strain JC94-2: *PHR1/PHR1*-*GFP* RP10::CIp20-*PHR1-GFP*) were grown in YPD-150 mM HEPES pH 8 at 25°C and analyzed by Confocal microscopy. *CHS3* null mutant expressing Phr1p-GFP (*chs3*Δ::*hisG*/*chs3*Δ::*hisG PHR1/PHR1*-*GFP* RP10::CIp20-*PHR1-GFP*) was analyzed by conventional fluorescence microscopy.

In a previous work we showed that Phr1p-GFP localizes to the septum in *C. albicans* cells at cytokinesis (Ragni et al., 2011). However, only one allele of *PHR1-GFP* was present in the strain used (strain 9.4). To improve the detection of Phr1p-GFP, we repeated the study using strain JC94-2 that contains two copies of *PHR1-GFP* and a wild type *PHR1* allele and whose construction is described elsewhere (Hopke et al., 2016). As expected, a bright fluorescence was detected at the bud periphery, whereas a very intense and thick fluorescent signal was present at the septum supporting the presence of the protein at the chitin ring and septum (Figure 1). The optical sections from a confocal microscope analysis indicated that Phr1p-GFP was distributed along the entire septum thickness (Supplementary Figure 1). To avoid the interference of the chitin ring that is deposited at the mother-bud neck by Chs3p, we analyzed the localization of Phr1p-GFP in cells lacking Chs3p. *C. albicans chs3*Δ cells showed a faint CF-staining except at the PS where a thin line at the center of the neck constriction was visible (Figure 1). In these cells, Phr1p-GFP signal was intense at both sides of the neck constrictions and along the line of the PS. These results indicate that Phr1p-GFP localizes to, or in position proximal to, the chitin disk, either in the secondary septa or in the plasma membrane sides facing the secondary septa and also at the neck constrictions despite the absence of the chitin ring.

**Figure 2:**
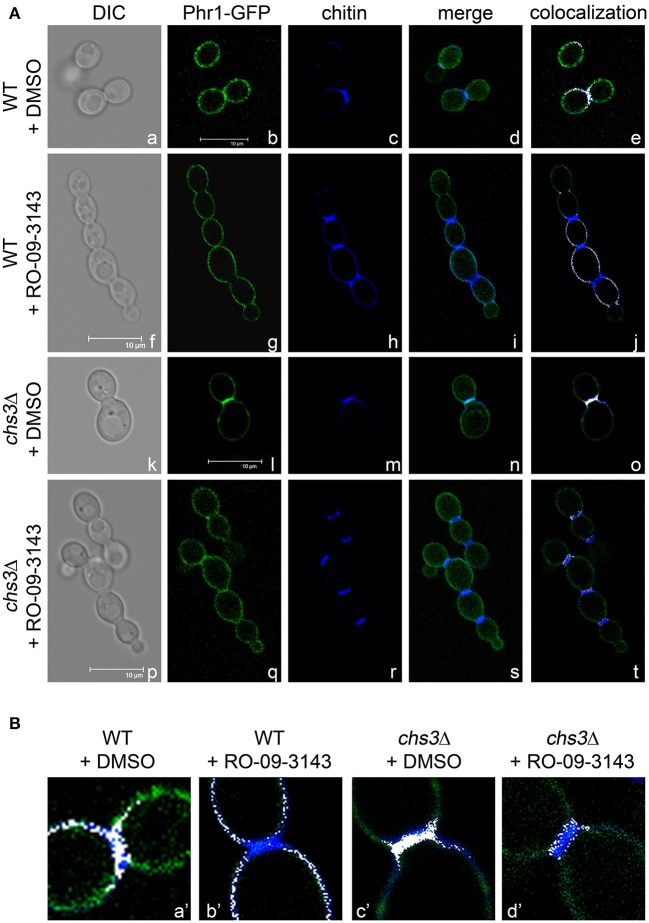
Inhibition of Chs1p alters the localization of Phr1p-GFP in the septum region. **(A)** Formation of alternative septa in wild type and *chs3*Δ cells grown in the presence of Chs1p inhibitor RO-09-3143 (10 μM). Images of Phr1p-fluorescence, CF fluorescence, their merge and the blue and green co-localization depicted in white are shown. **(B)** Magnification of the neck region. The merge of the CF-fluorescence and the green fluorescence (Phr1p-GFP) was artificially colored in white by ImageJ (NIH) and contrast adjusted in Photoshop CS5 (Adobe).

Next, we tested the effect of the inhibition of septum formation on the localization of Phr1p-GFP. Since Chs1p is an essential chitin synthase (Munro et al., 2001), we used RO-09-3143, a highly specific inhibitor of this enzyme (Sudoh et al., 2000). RO-09-3143, dissolved in DMSO, was added at the concentration of 10 μM to *C. albicans* exponentially growing cells (see section Growth and Viability in Yeast and Hyphal Forms in the Presence of the Chs1p Inhibitor). In DMSO-treated cells, Phr1p-GFP was detected at the usual sites (plasma membrane, bud periphery, and septum; Ragni et al., 2011). In cells at cytokinesis Phr1p-GFP co-localized with the septum region (Figure 2Aa–e and details in Figure 2Ba'). After 5 h of treatment with the drug, chains of 4–6 aligned cells were present and a thick CF-positive line separated the cells suggesting that an alternative chitin septum was produced (Figure 2Af–h). Interestingly, Phr1p-GFP was present at the cell periphery but did not localized to the new septa as little or no overlap of the green and blue signals was observed and no Phr1p-GFP co-localization with the chitin line was detected in the treated cells (Figure 2Ai,j and details in Figure 2Bb'). At 24 h of treatment, chains were long and branched and Phr1p-GFP localized to the plasma membrane but was not present in the alternative septa (data not shown). Thus, Phr1p-GFP is excluded from the septa produced in conditions of PS inhibition.

We also analyzed the localization of Phr1p-GFP in a *chs*3Δ mutant treated with DMSO (Figure 2Ak–o and detail in Figure 2Bc') or with RO-09-3143 (Figures 2Ap–t,Bd'). After 5 h of treatment, the chained cells showed the presence of a chitin-rich septum in which Phr1p-GFP was not detected (details in Figure 2Bd'). Thus, Phr1-GFP is excluded from the alternative septum both in the wild type or in the *chs3*Δ mutant (see further Discussion).

### Inhibition of Septum Formation Causes Destabilization of the Cell Wall and Defects in Nuclear Segregation in Cells Lacking β-(1,3)-Glucan Remodeling

#### Effect of RO-09-3143 During Growth in the Yeast Form

**Figure 3:**
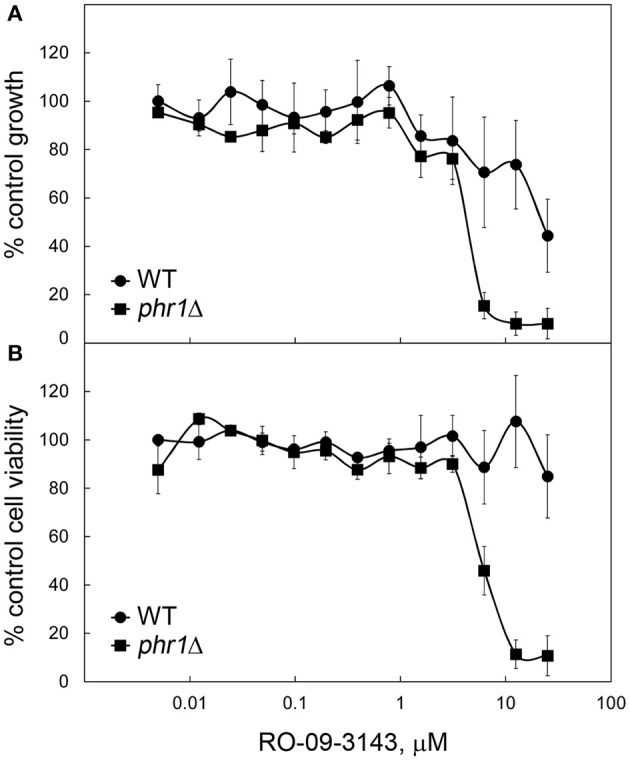
Higher susceptibility to RO-09-3143 of cells lacking β-(1,3)-glucan remodeling. **(A)** Susceptibility of a wild type (CAF3-1) and a *PHR1* null mutant (CAS8) to RO-09-3143 tested in YPD-pH 8 at 30°C in quadruplicates by broth microdilution assay at an inoculum size of 10^5^ cells/ml. Absorbance values are relative to the untreated wild type which was set to 100% (untreated wild type: mean A_595_ = 0.8498 ± 0.0585 SD; untreated *phr1*Δ = 0.8104 ± 0.0061 SD). **(B)** After reading the absorbance the plate was used to test cell viability by the XTT method. Data are relative to the absorbance value of the untreated wild type that was set to 100% (wild type A_490_ = 1.3655 ± 0.0281 SD; mutant: A_490_ = 1.1959 ± 0.1347 SD).

The susceptibility of the wild type strain and of a *PHR1* null mutant to RO-09-3143, was tested by broth microdilution assay (see section Broth Microdilution and XTT Assays). Due to the pH-dependent transcriptional pattern of *PHR1*, the phenotype of *phr1*Δ cells is manifested at neutral-alkaline pH values (Saporito-Irwin et al., 1995). Therefore, cells were grown in yeast form in YPD-150 mM HEPES, pH 8. As shown in Figure 3A, a *PHR1* null mutant was more sensitive to the drug compared to the wild type (MIC mutant ~ 6 μM; MIC wild type higher than 25 μM). XTT viability assay showed that the decrease of growth in the wild type was not associated to a decrease in viability suggesting that the drug is cytostatic on the wild type and cytocidal for the mutant (Figure 3B).

**Figure 4:**
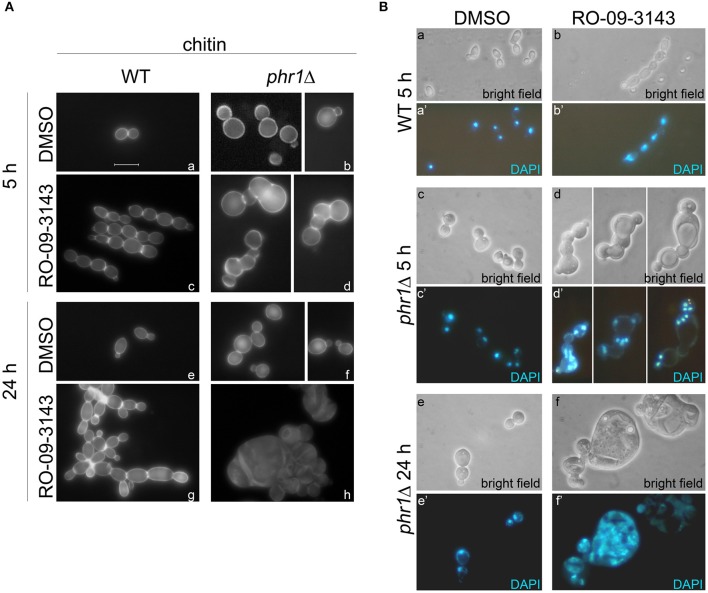
Effects of RO-09-3143 on cells lacking β-(1,3)-glucan remodeling. **(A)** Cell morphology examined by chitin staining of a wild type and *PHR1* null mutant treated at a cell density of 10^6^ cells/ml with DMSO (vehicle) or RO-09-3143 (10 μM) for the indicated time. Arrowheads indicate the abnormal enlargement of the constriction between cells in the treated *phr1*Δ mutant. **(B)** Nuclear staining of a wild type and *phr1*Δ mutant treated for 5 h as in **(A)**.

We analyzed in detail the effects of the inhibitor at a fixed concentration (10 μM) in batch cultures (see section Growth and Viability in Yeast and Hyphal Forms in the Presence of the Chs1p Inhibitor in Methods). Growth, measured as increase of OD_600_, proceeded in a parallel manner for the two strains and stopped after 8 h of treatment with the drug whereas the control cultures were unaffected for many hours (data not shown). DMSO-treated *phr1*Δ cells were rounder than wild type with wider bud necks and showed a tendency to aggregate (Saporito-Irwin et al., 1995). Cell morphologies and the presence of septa were visible in the CF-stained cells in Figure 4Aa,b. After 5 h of treatment with RO-09-3143, wild type cells appeared as linear chains of cells with CF-positive constrictions compared to the DMSO-treated cells between adjacent cells (Figure 4Ac) while the majority of *phr1*Δ mutant cells exhibited dramatic morphological changes such as formation of irregular and curved chains of cells, an abnormal enlargement of the bud neck, emergence of buds from random sites and lack of CF-stained alternative septa between the newly formed cells with consequent fusion of cytoplasm (Figure 4Ad). At 24 h of treatment, wild type chains of cells were branched with CF-positive constrictions compared to the DMSO-treated cells (Figure 4Ag) compared to the normal aspect of DMSO-treated cells (Figure 4Ae), whereas the majority of *phr1*Δ cells were 5–6 times larger than untreated *phr1*Δ (Figure 4Af), had long lines of ruptures with release of cellular material and appearance of cell ghosts, and were full of vacuoles and granules, indicating a total loss of cell integrity and morphology (Figure 4Ah).

Next, we analyzed the nuclear content by DAPI staining in DMSO- or RO-09-3143-treated wild type cells (Figure 4Ba,a',b,b') and phr1 mutant cells (Figure 4Bc,c',d,d'). At 5 h, *phr1*Δ treated cells contained two or more nuclei, reaching values of 6–7 nuclei in a single cell compartment, and nuclei were abnormally positioned. The increase in nuclei number seems to be a consequence not only of cytoplasm fusion but also of multiple endomitosis (EnM) (Figure 4Bd,d'). After 24 h of treatment, most of the treated *phr1*Δ cells were full of amorphous material and DNA, indicating that the majority of the cells lost integrity and died (Figure 4Bf,f') whereas DMSO-treated cells appeared viable and with the expected morphology (Figure 4Be,e'). As shown in Table 1, the majority of the wild type-treated cells had one nucleus per cell (Figure 4Bb,b'). On opposite, almost the totality of *phr1*Δ treated cells contained more than one nucleus in the same cell compartment and the class of cells devoid of a nucleus increased compared to the untreated culture indicating a failure to correctly segregate nuclei to the daughter cells (Table 1). Thus, upon inhibition of Chs1p activity, *phr1*Δ cells lose integrity especially at the neck region, undergo size increase, multiple EnM and aberrant nuclear segregation. To determine cell viability we performed a methylene blue (MB) permeability assay. MB-positive cells, or clusters with at least one blue cell/compartment, were counted as dead/lysed. After 5 h of treatment with RO-09-3143, 3% of wild type cells/chain and 6.8% of the mutant treated cells were MB-positive, respectively, compared to 2.3 or 5% of DMSO-treated wild type and mutant cells. At 24 h, the percentage of viable cells did not change in the wild type (9.4% for treated wild type cells and 1% for DMSO-treated cells) whereas dramatically increased in the mutant (83.5% for treated mutant cells compared to 3.9% for DMSO-treated cells). In conclusion, β-(1,3)-glucan branching/remodeling and Chs1p activity protect cells from abnormal enlargement, EnM and cell lysis.

**Table 1:** Distribution of nuclei in cells treated with the inhibitor of primary septum formation^a^.

	**1 Nucleus (%)**	**Nuclei ≥ 2 (%)**	**No nucleus (%)**
WT DMSO	97.74 ± 0.49	0.56 ± 0.26	1.69 ± 0.23
WT RO-09-3143	89.76 ± 5.45	3.77 ± 2.33	6.46 ± 3.12
*phr1*Δ DMSO	87.46 ± 2.71	8.06 ± 2.23	4.47 ± 0.49
*phr1*Δ RO-09-3143	4.08 ± 2.32	84.02 ± 0.75	11.90 ± 1.57

a *Cells were examined under the microscope with a double beam, bright field, and UV, to simultaneously monitor the cell morphology and the presence of DAPI-stained nucleus/i. The number of nuclei per single cell or per cell in a chain is reported. Cells without a visible nucleus were also counted and were the result of impaired nuclear segregation. A cell with the nucleus in mitosis correctly positioned at the neck between the mother and daughter cell was counted in the class 1 nucleus per cell*.

#### Effect of RO-09-3143 During Hyphal Development

After 5 h from induction of hyphal growth, wild type germ tubes developed into hyphae whereas *phr1*Δ germ tubes remained short and enlarged with wider septa and swollen aspect (Supplementary Figures 2a,b) as expected (Degani et al., 2016). In the presence of RO-09-3143, wild type hyphae were wider than untreated ones with enlarged hyphae apexes and constrictions (Supplementary Figure 2c). About 48% of the hyphal compartment had a single nucleus, 57% had more than one nucleus, and 5% had no nucleus, whereas in the DMSO-treated cells all the compartments contained one nucleus and compartments without nuclei were not detected. These data are consistent with those published on the effect of *CHS1* deletion (Munro et al., 2001). In the presence of RO-09-3143, *phr1*Δ mutant cells showed a worsening of the phenotype. Cells formed enlarged and branched short chains with constrictions and abnormal or poorly visible septa and two or more nuclei were detected in the same compartment (Supplementary Figure 2d). About 45% of the compartments had more than one nucleus and 19% had no nucleus, whereas in the untreated culture only about 1% underwent EnM (with no more than two nuclei in the same compartment) and no cell was without a nucleus. Similar results were obtained in two independent experiments.

In conclusion, RO-09-3143 induced mis-segregation of nuclei both in wild type and in *phr1*Δ null mutant but in the latter the percentage of compartments with no nuclei increased at the expenses of the category of compartments with more than 1 nucleus, indicating a worsening of the nuclear segregation defect.

At 24 h of treatment, DAPI entered in dead compartments only of the treated mutant and therefore a reliable count of nuclei was not possible (Supplementary Figures 2e–h).

## Discussion

Phr1p is required for cell wall biogenesis during the entire cell cycle and has a dynamic localization toward the sites of active cell wall growth. Moreover, Phr1p is likely required for the formation of high molecular weight glucan that confers mechanical strength to the bud neck as observed for *Sc*Gas1p (Cabib et al., 2012). In yeast cells, Fks1p concentrates at the division septum with a pattern equivalent to that shown for Gas1p and Phr1p (Utsugi et al., 2002). Phr1p is a new cytokinesis enzyme since the assembly of β-(1,3)-glucan synthesized by Fks1p, during/after invagination of the plasma membrane, and formation of the glucan-rich secondary septa on both sides of the of the chitinous disk (primary septum) requires the elongation and branching of this polysaccharide catalyzed by GH72 family of enzymes (Aimanianda et al., 2017).

In this work, we demonstrated that Phr1p localization at the septum is altered in conditions of primary septum inhibition and leads to the formation of alternative chitin-rich septa. Aberrant septa were previously identified in *S. cerevisiae chs2*Δ mutants that fail to form normal septa and divide, and produce thickened septa synthesized by *Sc*Chs3p (Schmidt et al., 2002; Cabib, 2004). While the normal septa have a typical tri-laminar structure, detectable also in *gas1*Δ mutant cells (Popolo et al., 1993), the aberrant septa are uni-laminar and chitin is deposited in a longitudinal instead of centripetal orientation with respect to the division plate line (Cabib, 2004). Aberrant septa were identified also in *C. albicans* (Walker et al., 2013). Previous studies have shown that chitin of the septum in cells treated with RO-09-3143 is deposited by the other chitin synthases (Chs3p, Chs2p or Chs8p). In *chs*3Δ cells treated with the inhibitor, Chs2p and Chs8p are the residual active chitin synthases, and are responsible for the formation of a type of alternative septa (Walker et al., 2013). The absence of β-(1,3)-glucan in these anomalous septa is likely the cause of the exclusion of Phr1p from these structure. These results support the notion that a tight coordination between the synthesis and remodeling of the β-(1,3)-glucan and PS formation exists.

The cell wall of *phr1*Δ cells is weakened but chitin increase operated by Chs3p compensates the defects and prevents cell lysis, as demonstrated by the severe swelling and lysis phenotype of a *phr1*Δ *chs3*Δ double mutant in vegetative growth at pH 8 (data not shown). The percentage of multinucleate cells in the double mutant was unaffected compared to the single mutants and cell lysis was the only predominant phenotypic trait (data not shown).

The inhibition of septum formation exhibited a different but strong phenotype in combination with *PHR1* deletion leading to abnormal enlargement of the neck constriction, and dramatic mis-segregation of the nuclei. These traits prevailed over lysis during the first 5 h of treatment with RO-09-3143. Moreover, the increase of cell mass and loss of cell shape at 24 h, support an additional role of Chs1p in cell integrity in agreement with a previous study (Munro et al., 2001).

*Schizosaccharomyces pombe* has a primary septum constituted of β-(1,3)-glucan. A cell wall β-(1,3)-glucan synthesized by a specific synthase is required to connect the cell wall with the plasma membrane and for contractile ring function at cytokinesis (Munoz et al., 2013; Cortes et al., 2015). We can speculate that Phr1p and Chs1p cooperate to confer rigidity to the cell wall at the membrane invagination site and permit a stable anchorage of AMR to cell wall and plasma membrane. In addition, the cortical region of the bud neck is a crucial site for formation of protein complexes involved in regulatory events leading to proper spindles positioning and cell division (Kusch et al., 2002). Thus, we can envisage that the weakening of the neck cell wall, brought about by the lack of Phr1p and inhibition of Chs1p, causes such severe perturbations that the spindle positioning checkpoint (SPOC) is not operative in arresting mitosis exit and consequently EnM, that are rarely present in normal cells, dramatically increase.

Since *phr1*Δ cells treated with the inhibitor showed lack/reduction of alternative septa, the formation of these septa seems a crucial process to protect cell viability. At this regard, caspofungin is fungistatic in *Aspergillus fumigatus* but becomes fungicidal if treatment is combined with the use of septum inhibitors (Dichtl et al., 2015).

In conclusion, Phr1p and Chs1p of *C. albicans* could be a combination of fungal-specific molecular targets useful for the development of innovative strategies in the fight against invasive fungal infections.

## Data Availability Statement

The raw data supporting the conclusions of this manuscript will be made available by the authors, without undue reservation, to any qualified researcher.

## Author Contributions

LP and GD designed the experiments and analyzed the data. GD performed the experiments and prepared tables and figures. LP wrote the manuscript that was read, discussed, and approved by GD.

### Conflict of Interest

The authors declare that the research was conducted in the absence of any commercial or financial relationships that could be construed as a potential conflict of interest.

## Supplementary Material

Supplementary Figure 1 Phr1p-GFP concentrates over the entire thickness of the septum. Cell (strain JC94-2) were grown at 25°C in YPD-150 mM HEPES buffered at pH 8. Elaboration by a Leica software provided the integration of image series in cross-sections along the X-Z-axis.

Supplementary Figure 2 Effects of RO-09-3143 on cells lacking β-(1,3)-glucan remodeling during hyphal development. Analysis of the effects of the inhibition of Chs1p on wild type **(a,c,e,g)** and *phr1*Δ mutant **(b,d,f,h)** during induction of hyphal growth in M199-buffered at pH 7.5 at 37°C. Micrographs of DAPI-stained cells were obtained by dual beam analysis, bright-field and UV light, to show simultaneously cell morphology and nuclei. Bar: 10 μm. Similar results were obtained in two independent experiments.
