# Supplementary figures and images for: The Glucan-Remodeling Enzyme Phr1p and the Chitin Synthase Chs1p Cooperate to Maintain Proper Nuclear Segregation and Cell Integrity in Candida albicans
Source: Front Cell Infect Microbiol. 2019 Nov 22;9:400. doi: 10.3389/fcimb.2019.00400 (PMC6882867; doi:10.3389/fcimb.2019.00400)

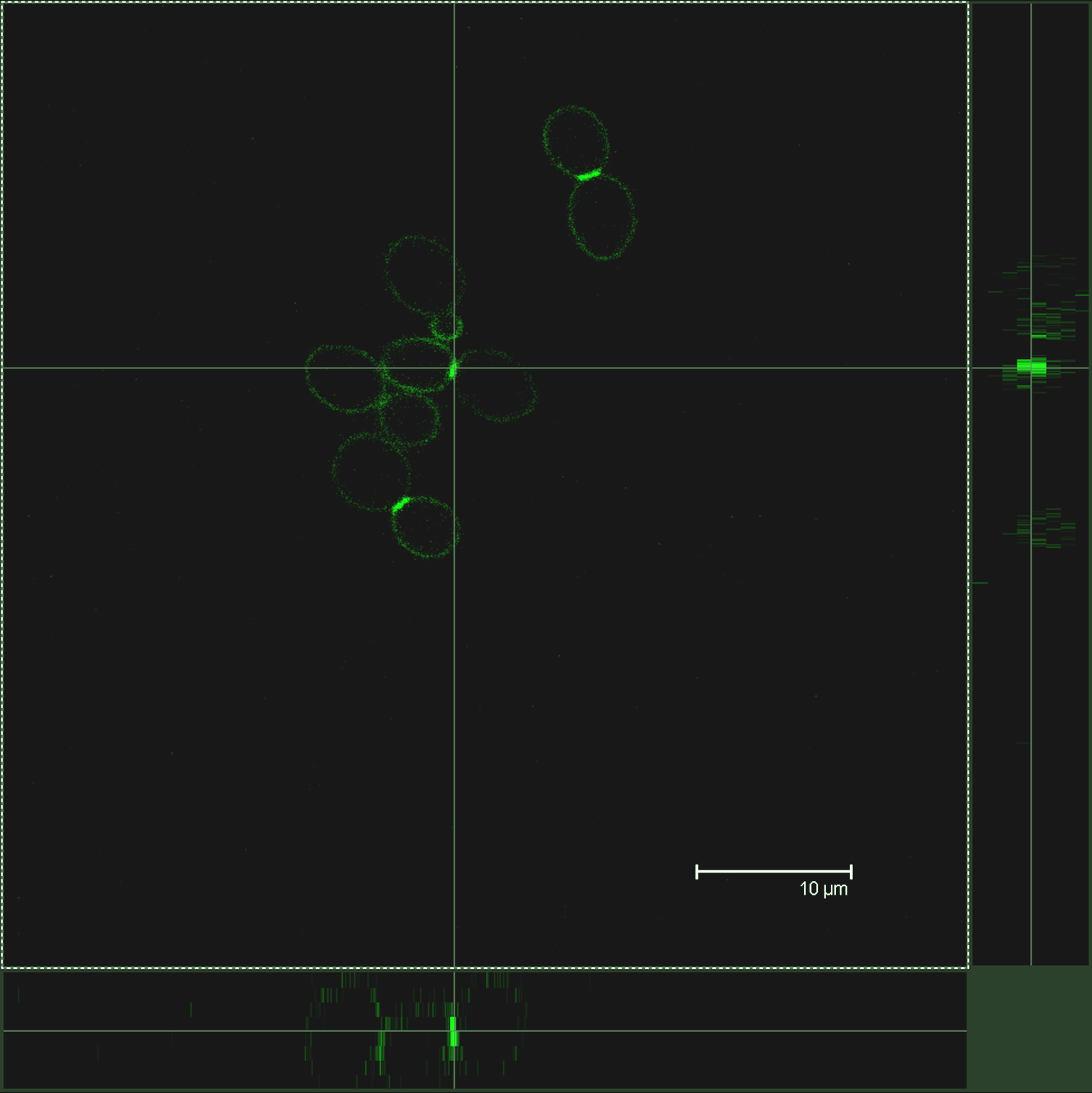

Supplement: Supplementary Figure 1 — Phr1p-GFP concentrates over the entire thickness of the septum. Cell (strain JC94-2) were grown at 25°C in YPD-150 mM HEPES buffered at pH 8. Elaboration by a Leica software provided the integration of image series in cross-sections along the X-Z-axis. [file Image_1.TIF]

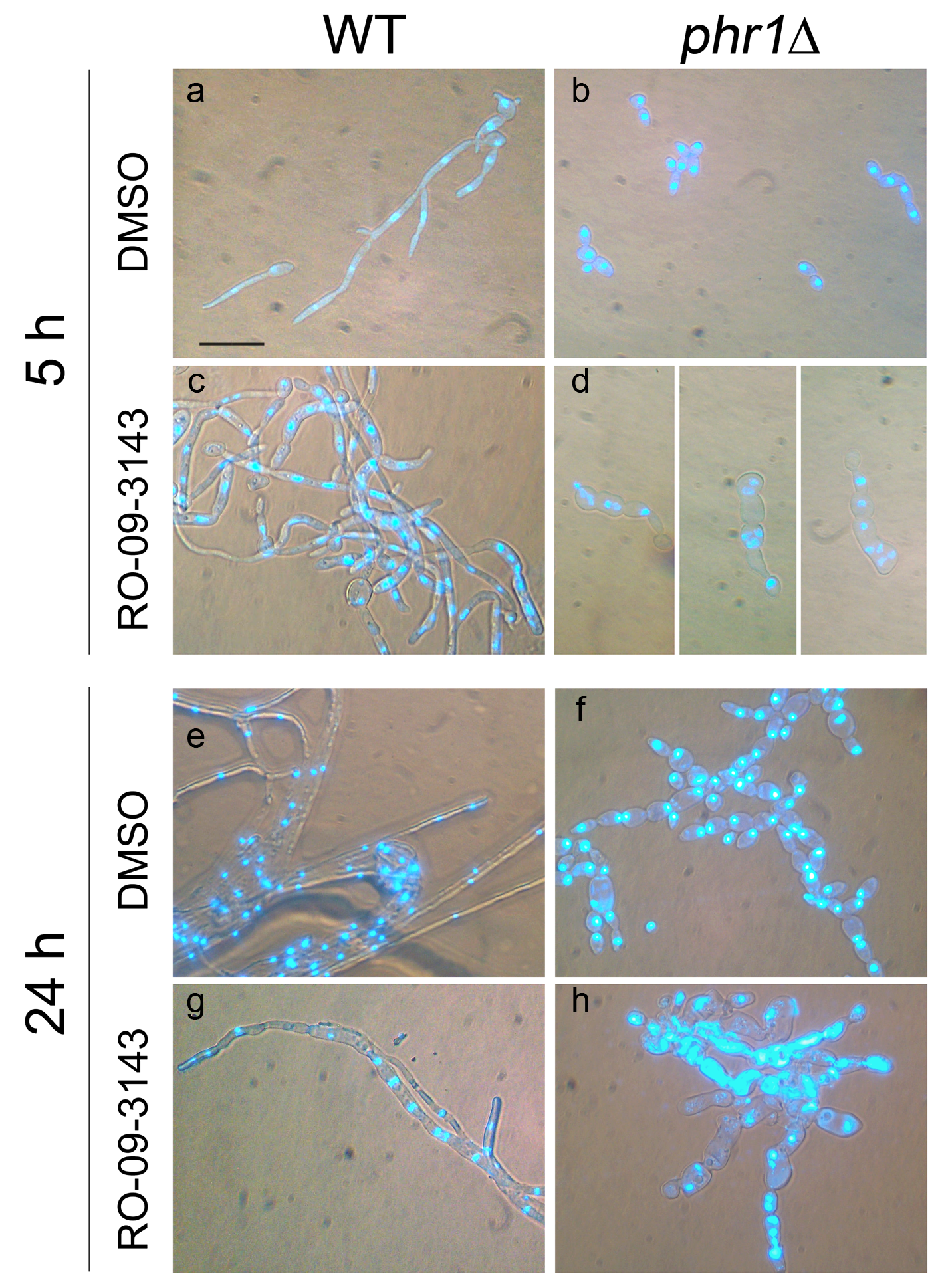

Supplement: Supplementary Figure 2 — Effects of RO-09-3143 on cells lacking β-(1,3)-glucan remodeling during hyphal development. Analysis of the effects of the inhibition of Chs1p on wild type (a,c,e,g) and phr1Δ mutant (b,d,f,h) during induction of hyphal growth in M199-buffered at pH 7.5 at 37°C. Micrographs of DAPI-stained cells were obtained by dual beam analysis, bright-field and UV light, to show simultaneously cell morphology and nuclei. Bar: 10 μm. Similar results were obtained in two independent experiments. [file Image_2.TIF]
